# A18 INTERKINGDOM DYNAMICS AND NUTRITION ARE ASSOCIATED WITH DIVERGENT MATURATIONAL PATTERNS OF THE INFANT BACTERIAL AND FUNGAL GUT MICROBIOME

**DOI:** 10.1093/jcag/gwac036.018

**Published:** 2023-03-07

**Authors:** E Mercer, M -C Arrieta, M Azad, E Simons, M Sears, T Moraes, P Mandhane, P Subbarao, S Turvey

**Affiliations:** 1 University of Calgary, Calgary; 2 University of Manitoba, Winnipeg; 3 McMaster University, Hamilton; 4 University of Toronto, Toronto; 5 University of Alberta, Edmonton; 6 University of British Columba, Vancouver, Canada

## Abstract

**Background:**

Early life has been identified as a critical window, during which time deviations from typical patterns of gut microbiome maturation have been associated with adverse health outcomes later in life. In the first 2-3 years of life, the infant gut microbiome undergoes ecological shifts characterized by increasing bacterial alpha diversity and variable changes in fungal alpha diversity. Research has shown not all infants follow these maturational trends, but our understandings of the factors linked to atypical microbiome maturation patterns are limited.

**Purpose:**

We assessed bacterial and fungal gut microbiome maturation in early life to determine if atypical maturational patterns were observed in otherwise healthy infants and identify factors associated with these patterns.

**Method:**

In 100 infants from the CHILD Cohort Study, we assessed the bacterial and fungal gut microbiome in stool samples collected at 3 and 12 months of age using 16S and ITS2 Illumina sequencing, respectively. We performed untargeted metabolomics on urine samples collected at 3 and 12 months using liquid chromatography-mass spectrometry/mass spectrometry. Microbiome and metabolomic measures were evaluated by ecological and multivariate analyses using RStudio.

**Result(s):**

Gut microbiome analyses revealed 24% and 20% of infants displayed atypical alpha diversity trajectories in the first year of life for bacteria or fungi, respectively. Atypical patterns were linked to reduced abundance of *Bacteroides* and increased *Candida* at 3 months. Functional analysis revealed an atypical bacterial alpha diversity trend was associated with elevated urinary trimethylamine N-oxide, creatine, indole acetic acid, and 2-furoylglycine, and an atypical fungal trend was associated with elevated urinary lactate. Using decision trees, the strongest predictors of atypical alpha diversity trends were interkingdom dynamics, breastfeeding duration, and maternal diet during pregnancy. Logistic regression revealed an atypical bacterial trend was positively associated with delivery via C-section and inversely associated with exclusive breastfeeding at 3 months, and an atypical fungal trend was positively associated with gestational consumption of artificially sweetened beverages and inversely associated with prenatal antibiotics. Interkingdom network analyses revealed the gut microbiome of infants with an atypical bacterial or fungal alpha diversity trend displayed a greater number of interkingdom interactions reflective of a less stable or immature gut microbiome.

**Conclusion(s):**

Our findings reveal a substantial proportion of infants display atypical patterns of gut microbiome maturation in the first year of life. While known microbiome-modifying factors were important determinants of maturational patterns, these factors were generally less influential than interkingdom influences. Together, this highlights the importance of interkingdom analyses at the individual level to generate more nuanced understandings of maturational trajectories in early life.

**Please acknowledge all funding agencies by checking the applicable boxes below:**

CIHR, Other

**Please indicate your source of funding;:**

Alberta Children's Hospital Research Institute

**Disclosure of Interest:**

None Declared

